# Our Response to COVID-19 as Endocrinologists and Diabetologists

**DOI:** 10.1210/clinem/dgaa148

**Published:** 2020-03-31

**Authors:** Ursula B Kaiser, Raghavendra G Mirmira, Paul M Stewart

**Affiliations:** 1 Department of Medicine, Brigham and Women’s Hospital, Boston, Massachusetts; 2 Department of Medicine, University of Chicago, Chicago, Illinois; 3 Faculty of Medicine and Health, University of Leeds, Leeds, UK

In our professional lives, we have not witnessed a healthcare crisis of this magnitude and severity. As we proof this (March 30, 2020), COVID-19, caused by the SARS-CoV-2 virus and labeled a global pandemic by the World Health Organization, is sweeping across the globe—over 730 000 cases and 35 000 deaths, with cases reported in over 190 countries. These numbers will rise substantially in forthcoming weeks and months.

We will all be in the midst of national and locally driven crisis management plans that understandably will impact our routine practice as we prioritize acute care to the most vulnerable. Over and above this, we felt it timely to highlight a few areas where our discipline-specific contribution can deliver a major impact.

First, for patients treated with glucocorticoids, it will be invaluable to reiterate “sick day rules” for our known patients with primary and secondary adrenal insufficiency taking glucocorticoid replacement therapy. As it relates to COVID-19, any patient with a dry continuous cough and fever should immediately double their daily oral glucocorticoid dose and continue on this regimen until the fever has subsided. Deteriorating patients and those who experience vomiting or diarrhea should seek urgent medical care and be treated with parenteral glucocorticoids (1).

More impactful will be the extension of these guidelines to the ~5% of patients in our populations taking chronic therapeutic corticosteroids by differing routes for underlying inflammatory conditions. The prevalence of adrenal insufficiency in these patients is high (~50%) irrespective of mode of delivery (2). Currently there is little evidence to guide us on when to intervene in terms of duration of prior corticosteroid exposure or on the impact of dose, either at a higher dose where supplemental steroid cover may not be necessary or a lower dose where adrenal suppression may not be as prevalent. In the interim, it seems logical, if not essential, that we identify all patients taking corticosteroids for whatever reason as high risk. We know from the published reports to date that these patients will be overrepresented in those at greatest risk of dying from COVID-19—the elderly and those with co-morbidities that include diabetes, hypertension, and chronic inflammatory disease (3,4). Moreover, those patients taking supraphysiologic doses of glucocorticoids may have increased susceptibility to COVID-19 as a result of the immunosuppressive effects of steroids, comorbidities of underlying immune disorders for which the steroids were prescribed, or immunomodulatory actions of other therapies prescribed in conjunction with glucocorticoids for the underlying disease. Reversing potential adrenal failure as a cause of mortality with parenteral glucocorticoid therapy is easy and simple to do once the issue has been recognized. The intent here is to ensure that no patient with a history of prior exposure to chronic glucocorticoid therapy (>3 months) by whatever route should die without consideration for parenteral glucocorticoid therapy. As a community, we will be key to ensuring recognition, management, and implementation of these important measures.

In this context, it will be important to communicate the reason underpinning glucocorticoid use. Based on prior experience in patients with acute respiratory distress syndrome and those affected with SARS and MERS (5), where glucocorticoid therapy was without benefit or associated with higher rates of invasive ventilation and mortality, the World Health Organization guidance is not to prescribe glucocorticoids (6). Physiological stress doses of hydrocortisone (50–100 mg intravenously t.i.d) not pharmacological doses of other corticosteroids should be given.

Second, the impact on patients with pituitary or other neuroendocrine disease also needs to be considered. As for patients with primary adrenal insufficiency, many of these patients have hypopituitarism including secondary adrenal insufficiency, requiring stress dose glucocorticoid supplementation as previously noted. Moreover, these patients may also have diabetes insipidus, further compounding fluid and electrolyte disorders and requiring careful monitoring and judicious water and electrolyte replacement to prevent hyponatremia or hypernatremia. This is particularly important in the context of increased insensible fluid loss associated with fever and tachypnea, combined with impaired ability for fluid intake with altered level of consciousness (7).

Third, for patients with diabetes mellitus, whereas the risk of contracting a viral illness is no greater than those without diabetes mellitus, severity of disease from viral infections is notably greater. Recent published reports from the Wuhan province in China (3,4) reveal that those with diabetes mellitus and hypertension were overrepresented among the most severely ill patients with COVID-19 and those succumbing to the disease. Whether this susceptibility to illness severity is especially greater in the case of COVID-19 or simply a reflection of the greater risk posed by diabetes remains uncertain at this point. Current guidance from the Centers for Disease Control and Prevention for prevention of COVID-19 for those with diabetes is no different than the general population, but the recognition that diabetes poses a greater risk for severity of illness should prompt health-care providers to be more vigilant in the assessment of such patients who present with concerning symptoms (ie, shortness of breath, fever) (8).

Finally, as clinician scientists, we realize that research and innovation will ultimately provide solutions to this crisis, whether through enhanced diagnostics, innovative therapies, or future vaccines. A potentially exciting endocrine-connected observation is the elucidation of the mechanism of entry of SARS-CoV-2 into cells. Here, angiotensin-converting enzyme 2 (ACE2) is now established as the SARS-CoV receptor (9) but with conflicting data as to its translational relevance. It has been suggested that angiotensin-converting enzyme inhibitors/angiotensin receptor blockers might increase susceptibility and severity to COVID-19 through upregulation of ACE2 and thereby possibly explain the overrepresentation of hypertensive patients in patients dying from COVID-19 (10). Upregulation of ACE2 might also explain the poor outcome in smokers versus nonsmokers, but it is important to stress that these are preliminary reports and should not result in changing prescribed medications at this stage (11). APN01 is a recombinant human ACE2 developed by APEIRON for the treatment of acute lung injury, acute respiratory distress syndrome, and pulmonary arterial hypertension; by slowing viral entry into cells and viral spread, it may be beneficial, and clinical trials are underway (12). Conversely, angiotensin II is known to stimulate alveolar epithelial cell apoptosis, and inhibition of this with angiotensin receptor 1 blockers such as losartan might reduce mortality from acute respiratory distress syndrome in COVID-19 infection (13).

Perhaps justifying greater excitement is the downstream transmembrane protease serine 2 required for SARS-CoV-2 viral spike protein priming and onward transmission (14). Camostat mesylate, a transmembrane protease serine 2 inhibitor, has been approved in Japan for the treatment of pancreatic inflammation and when tested on SARS-CoV-2 isolated from a patient prevented the entry of the virus into lung cells. Endocrine-related targets are at the forefront of discovery science as we collectively tackle this pandemic.
